# Iron chelation and redox chemistry of anthranilic acid and 3-hydroxyanthranilic acid: A comparison of two structurally related kynurenine pathway metabolites to obtain improved insights into their potential role in neurological disease development

**DOI:** 10.1016/j.jorganchem.2015.01.005

**Published:** 2015-04-15

**Authors:** Vladimir Chobot, Franz Hadacek, Wolfram Weckwerth, Lenka Kubicova

**Affiliations:** a Division of Molecular Systems Biology, Department of Ecogenomics and Systems Biology, Faculty of Life Sciences, University of Vienna, Althanstrasse 14, Vienna A-1090, Austria; b Plant Biochemistry, Albrecht-von-Haller Institut, Georg-August-Universität Göttingen, Justus-von-Liebig-Weg 11, Göttingen D-37077, Germany

**Keywords:** Iron coordination complexes, Reactive oxygen species, Kynurenines, Differential pulse voltammetry, Mass spectrometry, 3-Hydroxyanthranilic acid:anthranilic acid ratio

## Abstract

Anthranilic acid (ANA) and 3-hydroxyanthranilic acid (3-HANA) are kynurenine pathway intermediates of the tryptophan metabolism. A hitherto unemployed method combination, differential pulse voltammetry, mass spectrometry (nano-ESI–MS), deoxyribose degradation and iron(II) autoxidation assays has been employed for studying of their redox chemistry and their interactions with iron(II) and iron(III) ions. Both acids inhibited the Fenton reaction by iron chelation and ROS scavenging in the deoxyribose degradation assay. In the iron(II) autoxidation assay, anthranilic acid showed antioxidant effects, whereas 3-hydroxyanthranilic acid exhibited apparent pro-oxidant activity. The differential pulse voltammograms of free metabolites and their iron(II) coordination complexes reflected these properties. Nano-ESI–MS confirmed ANA and 3-HANA as efficient iron(II) chelators, both of which form coordination complexes of ligand:iron(II) ratio 1:1, 2:1, and 3:1. In addition, nano-ESI–MS analyses of the oxidation effects by hydroxyl radical attack identified 3-HANA as strikingly more susceptible than ANA. 3-HANA susceptibility to oxidation may explain its decreased concentrations in the reaction mixture. The presented observations can add to explaining why 3-HANA levels decrease in patients with some neurological and other diseases which can often associated with elevated concentrations of ROS.

## Highlights

• Anthranilic (ANA) and 3-hydroxyanthranilic acid (3-HANA) are tryptophan metabolites.
• ANA and 3-HANA levels change in patients suffering from some neurological diseases.
• Oxidative hydroxyl radical attack affects 3-HANA more than ANA.
• Both acids can serve as ligands of transition metals and affect their redox cycling.
• These findings might explain why 3-HANA levels decrease in these patients.

## Graphical abstract

Anthranilic and 3-hydroxyanthranilic acid are tryptophan metabolites. Their redox chemistry and iron chelation were investigated.

**Figure:**
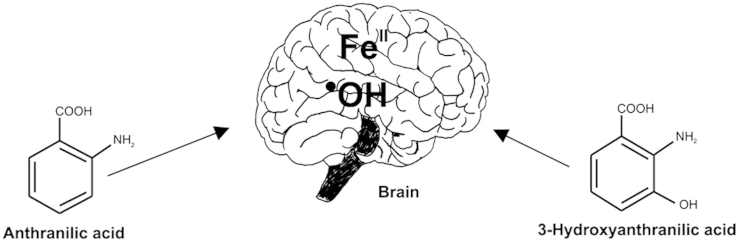

## Introduction

**Fig. 1:**
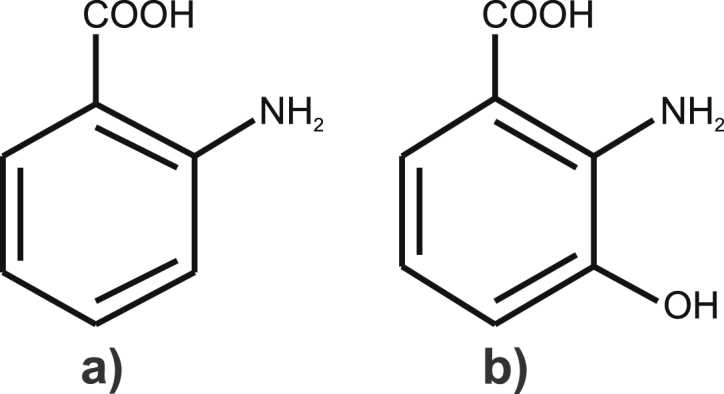
Structures of a) anthranilic acid (ANA) and b) 3-hydroxyanthranilic acid (3-HANA).

Anthranilic acid (2-aminobenzoic acid, ANA) and 3-hydroxyanthranilic acid (3-HANA) (Fig. 1) are metabolic intermediates of the kynurenine pathway starting from tryptophan. This pathway is responsible primarily for the biosynthesis of nicotinamide that is required for biosynthesis of NAD(P)H, a fundamental cellular electron donator [1]. In plants, ANA and 3-HANA are used for quinoline and acridine alkaloid synthesis [2]. In pharmaceutical industry, ANA served as a basic model compound for synthesis of fenamates (a group of anti-inflammatory drugs).

During the last decades, ANA and 3-HANA have been recognized to affect brain and immune system function. Patients with neurological disorders usually show marked changes in the levels of these two tryptophan metabolites [3], [4], [5]. 3-HANA concentration as well as the 3-HANA:ANA ratio decrease in patients suffering from stroke, chronic brain injury and Huntington's disease, among others [6], [7]. ANA and 3-HANA can influence ROS concentrations by formation of coordination complexes with iron [3]. In the human brain, ROS may arise as a product of iron(II) autoxidation and the Fenton chemistry [8], [9]. Whereas ANA is nearly exclusively known for antioxidant activity [3], [10], 3-HANA's documented activities include both antioxidant [3], [10], [11], [12] and pro-oxidant effects [3], [13], [14], [15]. So far, the mechanism behind the reversed 3-HANA:ANA ratio in patients is not understood well.

Although the redox and chelation properties of both acids have been investigated extensively in the last decades, some studies demonstrated controversial results. This stimulated us to explore these two specific tryptophan metabolites in more detail by employing a set of methods that have not yet been used in this combination in previous studies: differential pulse voltammetry, and direct infusion mass spectrometry (nano-ESI–MS), deoxyribose degradation and iron(II) autoxidation assays.

## Material and methods

### Chemicals

All chemicals used were purchased from Sigma–Aldrich (Schnelldorf, Germany). Water had Milli-Q quality.

### Differential pulse voltammetry

Voltammetric curves were recorded in a three-electrode system, μAutolab PGSTAT type III (EcoChemie Inc., Utrecht, The Netherlands). The working electrode was a glassy carbon electrode of 3 mm diameter, an Ag/AgCl (saturated KCl) electrode was used as reference, and platinum wire as a counter electrode. The glassy carbon electrode was washed with water and then polished by aluminium oxide powder (0.3 μm of grain size) before every measurement. The effective scan rate of the voltammetry was 21 mV s^−1^, step potential was 5.25 mV, modulation amplitude was 25 mV, modulation time was 50 ms, and interval time was 250 ms. Scan potential was from −500 to +1200 mV. FeSO_4_ was dissolved in degassed water at a concentration 10 mM. The stock solutions of the measured acids were prepared by dissolving in degassed buffer (0.1 M phosphate buffer pH 7.4). The ionic strength of the buffer was 1 M and it was adjusted by K_2_SO_4_. The samples for the electrochemical measurements of the acids were prepared by mixing of 1 mL their stock solution with 8 mL of the buffer and 1 mL of water. The final concentration of the acid was 1 mM. The samples for the coordination complex analysis were prepared by mixing 1 mL of aqueous FeSO_4_ solution with 9 mL of the degassed buffer or buffer–anthranilic or 3-HANA solution. The final applied concentration ratio of acid:Fe was 4:1. The solutions of electrolytes were degassed by argon for 10 min and measurements were carried out under argon atmosphere at a room temperature.

### Mass spectrometry analyses

MS analyses were performed out on a Thermo Electron LTQ-Orbitrap XL mass spectrometer equipped with a nano electrospray ion source (ThermoFisher Scientific, Bremen, Germany) and operated under Xcalibur software, in the positive ionization mode. The instrument was calibrated using the manufacturer's calibration standards. The Fourier transformed full scan mass spectra were acquired at a target value of 10^6^ ions with a resolution of 100,000 in the *m/z* range of 80–2000. In order to achieve even higher mass accuracy, a lock mass option was enabled and the cyclomethicone N5 ions generated in the electrospray process from ambient air (*m/z* = 371.101230) were used for internal recalibration in real time. This allowed mass accuracies of <1 ppm. Specific tune settings for the MS were as follows: spray voltage was set to 1.8 kV; capillary voltage was 45 V, tube lens offset 150 V and capillary temperature was set at 180 °C, no sheath gas and auxiliary gas were used. ANA and 3-HANA coordination complexes were measured according to a modified method that was published previously by Sarowar et al. [16]. The samples for the nano-ESI–MS analysis of ANA and 3-HANA– Fe^II^ complexes were prepared from a 1 mM stock solution of ANA or 3-HANA in degassed methanol by adding of appropriate amount of degassed aqueous FeCl_2_ solution (500 μM) in the (ANA or 3-HANA: Fe) molar ratios 1:1, 2:1, 3:1, and 4:1. Before the measurement, the samples were diluted 1:10 or 1:100 with water/methanol (50:50, v:v) mixture. For the nano-ESI–MS measurement, gold coated glass emitters (DNU-MS GbR, Berlin, Germany) with 5 μL of this final sample were used. The samples for nano-ESI–MS analyses of anthranilic and 3-hydroxyanthranilic acid oxidation stability were prepared as followed: The tested compound was dissolved in aqueous ammonium bicarbonate buffer solution (10 mM, pH 7.4 adjusted with HCl) to 1 mM solution. To 500 μL of this solution, 300 μL of 10 mM aqueous ammonium bicarbonate buffer, 100 μL of degassed aqueous FeCl_2_ solution (100 μM) and 100 μL of aqueous H_2_O_2_ solution (55 mM) were added. Standard 1.5 mL sample vials (La-Pha-Pack, Werner Reifferscheidt GmbH, Langerwehe, Germany) were used as reaction vials. The reaction mixtures was incubated at 27 °C and analyzed in reaction times 3 and 8 h. For the nano-ESI–MS measurement, 50 μL of the reaction mixture was diluted with 450 μL of 0.2% (v/v) formic acid in methanol and glass emitters (DNU-MS GbR, Berlin, Germany) filled with 5 μL of this mixture were used. The isotope exact masses of the complex ions and all other designated ions were calculated with ChemDoodle 7.0.2, iChemLabs, LLC, Somset, NY.

### Deoxyribose degradation assay

The deoxyribose degradation assay and its various variants follow published procedures [17]. ANA or 3-HANA was dissolved in aqueous KH_2_PO_4_/KOH buffer solution (30 mM, pH 7.4) and diluted serially; to 125 μL of this solution, 25 μL of a 10.4 mM 2-deoxy-d-ribose solution in the same buffer system and 50 μL of FeCl_3_ (FeCl_3_ variant) or Fe^III^EDTA (Fe^III^EDTA variant) solution (50 μM) were added. The complex of Fe^III^ with EDTA was prepared separately; the 104 μM EDTA solution in the buffer was premixed with the aqueous 100 μM FeCl_3_ solution (1:1, v/v). Further, 25 μL of 10.0 mM aqueous solution H_2_O_2_ and 25 μL of 1.0 mM ascorbic acid in the buffer were added to start the Fenton reaction in the H_2_O_2_/Fe^III^/ascorbic acid reaction mixture. In the other deoxyribose degradation assay systems, H_2_O_2_ or ascorbic acid was replaced by the same volume of water or buffer, respectively. The final concentrations of the tested acids were 2–500 μM. Thiobarbituric acid reactive species (TBARS) were determined photometrically at 532 nm after reaction with thiobarbituric acid and subsequent extraction of the pink pigment with 1-butanol. The H_2_O_2_/Fe^III^/ascorbic acid reaction mixture served as positive control and represented 100% TBARS detection in all variants and also served as comparative standard for each experiment. Blanks contained the full reaction mixtures except for 2-deoxy-d-ribose and were determined in each experiment. Experiments were performed in triplicates. The temperature during incubation was 27 °C. Variants containing H_2_O_2_ were evaluated after 1 h; variants without H_2_O_2_ were evaluated after 16 h incubation.

### Fe^II^ autoxidation assay

The assay was carried out according procedures published elsewhere [18], [19]. The sample was dissolved in aqueous KH_2_PO_4_/KOH buffer solution (30 mM, pH 7.4) and diluted serially; to 125 μL of this solution, 25 μL of a 52 mM 2-deoxy-d-ribose solution in the same buffer system, 50 μL of the buffer, and 50 μL of degassed aqueous FeSO_4_ solution (50 μM) were added. The final concentrations of the tested compounds were 2–500 μM. Blanks contained the full reaction mixtures except for 2-deoxy-d-ribose. Standard 1.5 mL sample vials (La-Pha-Pack, Werner Reifferscheidt GmbH, Langerwehe, Germany) were used as reaction vials. The mixtures were incubated at 27 °C for 16 h. Thereafter, 250 μL of 1.0% thiobarbituric acid dissolved in 3% trichloroacetic acid (w/v) was added to each vial to detect TBARS. The vials were heated in a water bath at 80 °C for 30 min. The reaction was stopped by transferring the vials into an ice water bath for 3 min. To extract the TBARS, 600 μL of 1-butanol was added, and the mixture was rigorously vortexed. The butanol layers of the vials, each 350 μL, were pipetted into flat bottomed 96 well plates (Greiner, Kremsmünster, Austria), and the absorbance was determined with a microplate reader (Tecan Infinite M200, Männedorf, Switzerland) at 532 nm. Experiments were performed in triplicate. Reaction mixtures lacking the test compound served as the positive control (100% TBARS). The phosphate buffer and water, which were used as solvents for the tested substances or FeSO_4_, were degassed by argon for 10 min at least.

### Statistical analysis

Statgraphics Centurion XVI (Statistical Graphics Corp., Rockville, MD, USA) was used to perform analyses of variance (ANOVA) employing 95% Duncan's multiple range *post hoc* test.

## Results and discussion

**Fig. 2:**
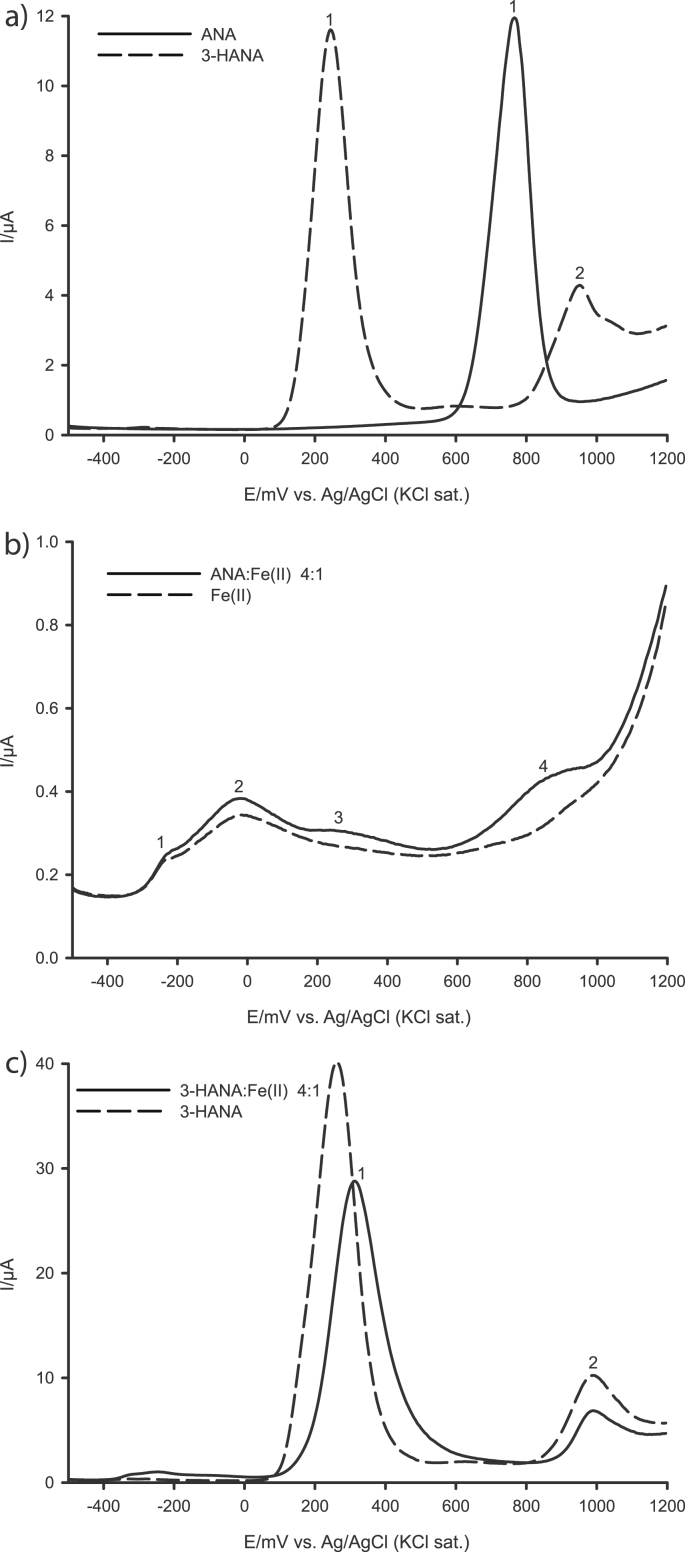
Differential pulse voltammetry of a) anthranilic acid (ANA) and 3-hydroxyanthranilic acid (3-HANA), b) anthranilic acid−iron(II) solution 4:1 and c) 3-hydroxyanthranilic acid−iron(II) solution 4:1.

The differential pulse voltammogram (DPV) of ANA showed one peak at 766 mV (peak 1), corresponding to oxidation of the amino group (Fig. 2a), and 3-HANA two peaks, at 242 and 947 mV corresponding to the hydroxyl group and amino group oxidations, respectively (Fig. 2a). The voltammetry proved that both tested acids are redox active compounds. The more negative redox potential of the 3-HANA phenolic hydroxyl group suggests that it is the more potent reducing agent of the two compounds. *Ortho*-aminophenols, such as 3-HANA, usually exhibit redox activity because of their easy oxidation to quinone imines [15], [20].

In addition, ANA and 3-HANA were investigated for their possible capability of forming coordination complexes with iron(II). The voltammogram demonstrated that iron(II) ions formed various coordination complexes with phosphate (buffer component), which resulted in a broad dominant peak with two maxima at the potentials at −235 mV (peak maximum 1) and −18 mV (peak maximum 2) (Fig. 2b). When iron(II) solution was added to the ANA solution, a new broad peak at 278 mV (peak 3, Fig. 2b) appeared in the voltammogram; the peak current of the amino group oxidation evidently decreased and the peak oxidation potential increased to 833 mV (peak 4). In the case of 3-HANA, no peaks of Fe−3-HANA complexes were visible (Fig. 2c). However, the oxidation peak of the hydroxyl group was shifted into the anodic direction (peak 1). The peak potential of the amino group oxidation remained unchanged (peak 2). A possible explanation for this phenomenon could be that many different iron coordination complexes with 3-HANA as ligand are formed, all of which were present in too low concentrations to allow unambiguous discrimination from the baseline.

**Fig. 3:**
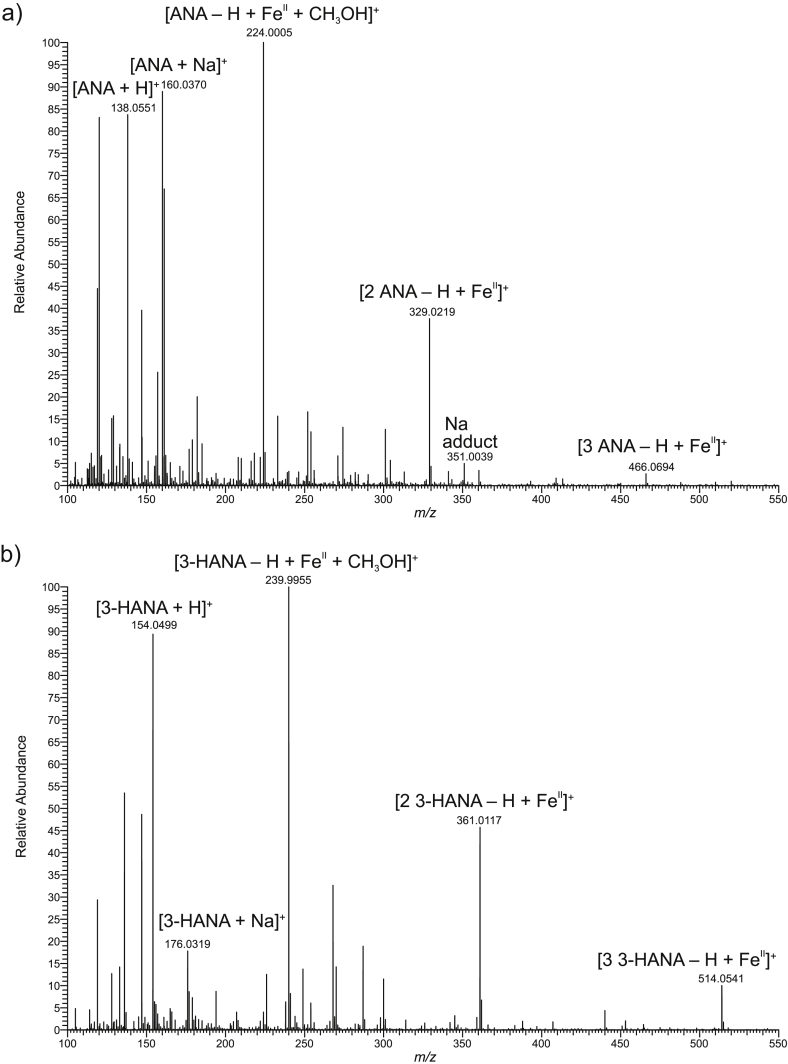
ESI–MS spectra in the positive mode of a) anthranilic acid (ANA)−iron(II) solution 4:1 and b) 3-hydroxyanthranilic acid (3-HANA)−iron(II) solution 2:1.

Consequently, coordination complex formation of ANA and 3-HANA with iron(II) was explored by nano-ESI–MS (Fig. 3). This method is applicable for characterizing metal complexes because it provides direct information about possible stoichiometry of metal coordination complexes [16], [21], [22]. The ESI–MS spectra proved the capability of ANA and 3-HANA to form coordination complexes with iron(II). The nano-ESI–MS measurements were carried out with solutions of ANA or 3-HANA with iron(II) ions. The mass spectrum of iron(II)−ANA solution (Fig. 3a) showed the expected peaks of ANA ([M + H]^+^, *m/z* 138.0551 (calc. *m/z* 138.0549) and *m/z* 160.0370 [M + Na]^+^ (calc. *m/z* 160.0370).

An ion detected at *m/z* 224.0005 (C_8_H_10_^56^FeNO_3_, calc. *m/z* 224.0005) corresponds to a coordination complex ion with one ANA ligand [M−H + Fe^II^ + CH_3_OH]^+^. The following iron isotopes were detectable: C_8_H_10_^54^FeNO_3_, *m/z* 222.0053, calc. *m/z* 222.0052, rel. int. 6%, calc. rel. int., 6%; C_8_H_10_^56^FeNO_3_, rel. int. 100%, calc. rel. int. 100%; C_8_H_10_^57^FeNO_3_, *m/z* 225.0039, calc. *m/z* 225.0038, rel. int. 7%, calc. rel. int. 9%; C_8_H_10_^58^FeNO_3_, *m/z* 226.0044, calc. *m/z* 226.0043, rel. int. 0.2%, calc. relative int. 0.2%.

A further metal complex ions was detected at *m/z* 329.0219 that corresponds to [2M−H + Fe^II^]^+^, C_14_H_13_^56^FeN_2_O_4_, calc. *m/z* 329.0219. The following iron isotope ions were detectable: C_14_H_13_^54^FeN_2_O_4_, 327.0267, calc. 327.0266, rel. int. 6%, calc. rel. int. 6%; C_14_H_13_^56^FeN_2_O_4_, rel. int. 100%, calc. rel. int. 100%; C_14_H_13_^57^FeN_2_O_4_, *m/z* 330.0254, calc. rel. int. 330.0252, rel. int. 14%, calc. rel. int. 15%; C_14_H_13_^58^FeN_2_O_4_, *m/z* 331.0263, calc. *m/z* 331.0262, rel. int. 0.3%, calc. rel. int. 0.3%.

An *m/z* 351.0039 (C_14_H_12_^56^FeN_2_NaO_4_, calc. *m/z* 351.0038) represented the Na adduct of the former complex [2M–2H + Fe^II^ + Na]^+^. The following iron isotope ions were detectable: C_14_H_12_^54^FeN_2_NaO_4_, *m/z* 349.0087, calc. *m/z* 349.0085, rel. int. 5%, calc. int. 6%; C_14_H_12_^56^FeN_2_NaO_4_, rel. int. 100%, calc. int. 100%; C_14_H_12_^57^FeN_2_NaO_4_, *m/z* 352.0071, calc. *m/z* 352.0073, rel. int. 14%, calc. rel. int. 15%, C_14_H_12_^58^FeN_2_NaO_4_, *m/z* 353.0055, calc. *m/z* 353.0057, rel. int. 0.3%, calc. rel. int. 0.3%.

A further ion detected at *m/z* 466.0694 was assigned to C_21_H_20_^56^FeN_3_O_6_, [3M−H + Fe^II^]^+^, calc. *m/z* 466.0693. The following iron isotope ions were detectable: C_21_H_20_^54^FeN_3_O_6_, *m/z* 464.0742, calc. *m/z* 464.0739, rel. int. 4%, calc. rel. int. 6%; C_21_H_20_^56^FeN_3_O_6_, rel. int. 100%, calc. rel. int. 100%; C_21_H_20_^57^FeN_3_O_6_, *m/z* 467.0726, calc. *m/z* 467.0727, rel. .int. 20%, calc. rel. int. 22%; C_21_H_20_^58^FeN_3_O_6_, *m/z* 468.0734, calc. *m/z* 468.0732, calc. rel. int. 0.5%.

In the ESI–MS spectrum of iron(II)−3-HANA solution, a prominent peak at *m/z* 154.0499 was identified as the protonated ion of 3-HANA [M + H]^+^, calc. 154.0500. (Fig. 3b). The 3-HANA adduct with sodium ([M + Na]^+^) appeared as *m/z* 176.0319 signal (calc. 176.0318).

The first metal complex was assigned to the ion [M−H + Fe^II^ + CH_3_OH]^+^, C_8_H_10_^56^FeNO_4_, *m/z* 239.9955, calc. *m/z* 239.9955. The following iron isotope ions were detectable: C_8_H_10_^54^FeNO_4_, *m/z* 238.0002, calc. *m/z* 238.0000, rel. int. 6%, calc. rel. int. 6%; C_8_H_10_^56^FeNO_4_, rel. int. 100%, calc. rel. int. 100%; C_8_H_10_^57^FeNO_4_, *m/z* 240.9988, calc. *m/z* 240.9987, rel. int. 8%, calc. rel. int. 8%); C_8_H_10_^58^FeNO_4_, *m/z* 241.9994, calc. *m/z* 241.9996, rel. int. 0.5%, calc. rel. int. 0.8%.

A further metal complex ion with two 3-HANA ligands had an *m/z* 361.0117 that correspond to [2M−H + Fe^II^]^+^, C_14_H_13_^56^FeN_2_O_6_, calc. *m*/*z* 361.0117. The following iron isotope ions were detectable: C_14_H_13_^54^FeN_2_O_6_, *m/z* 359.0165, calc. *m/z* 359.0164, rel. int. 6%, calc. rel. int. 6%; C_14_H_13_^56^FeN_2_O_6_, rel. int. 100%, calc. rel. int. 100%; C_14_H_13_^57^FeN_2_O_6_, *m/z* 362.0151, calc. *m/z* 362.0150, rel. int. 15%, calc. rel. int. 15%; C_14_H_13_^58^FeN_2_O_6_, *m/z* 363.0189, calc. *m/z* 363.0185, rel. int. 0.7%, calc. rel. int. 1%.

A metal complex ion with three 3-HANA ligands was identified at *m/z* 514.0541, corresponding to C_21_H_20_^56^FeN_3_O_9_, [3M−H + Fe^II^]^+^, calc. *m/z* 514.0541. The following iron isotope ions were detectable: C_21_H_20_^54^FeN_3_O_9_, *m/z* 512.0588, calc. *m/z* 512.0588, rel. int. 3%, calc. int. 6%; C_21_H_20_^56^FeN_3_O_9_, rel. int. 100%, calc. rel. int. 100%; C_21_H_20_^57^FeN_3_O_9_, *m/z* 515.0576, calc. *m/z* 515.0574, rel. int. 18%, calc. rel. int. 22%; C_21_H_20_^58^FeN_3_O_9_, *m/z* 516.0578, calc. *m/z* 516.0583, rel. int. 2%, calc. rel. int. 2%.

**Fig. 4:**
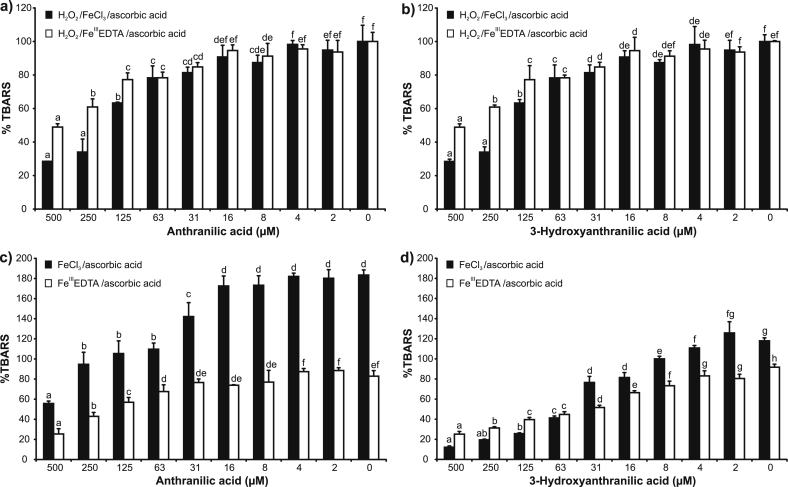
Effects of anthranilic acid and 3-hydroxyanthranilic acid on generation of thiobarbituric acid reactive species in variants of deoxyribose degradation assay. EDTA, ethylenediaminetetraacetic acid. Error bars indicate the standard deviation of three replicates; letters (a–h) indicate different levels of significance (95% Duncan).

All ions mentioned above were detected with different intensities in ANA or 3-HANA: Fe molar ratios 1:1, 2:1, 3:1 and 4:1 (data not shown). These observations were corroborated by the deoxyribose degradation assay results (Fig. 4). The principal chemical reaction of the assay is the oxidative degradation of 2-deoxy-d-ribose by hydroxyl radicals arising in the Fenton reaction [17], [23], [24]. The oxidation of 2-deoxy-d-ribose leads to the production of thiobarbituric acid reactive species (TBARS). The reaction mixture contains hydrogen peroxide, iron(III) and ascorbic acid. Ascorbic acid starts the Fenton reaction by reduction of iron(III) to iron(II). Iron is added either as FeCl_3_ or in complex with ethylenediaminetetraacetic acid (EDTA). Ascorbic acid is used because it is known as a common cellular reducing agent. If iron ions are added as FeCl_3_, they can form complexes with the test compound, as EDTA complex this is less likely to occur. Further modifications of this assay, for example if hydrogen peroxide and/or ascorbic acid are not added, provide additional information about the redox chemistry of the test compounds [17]. The variants of the deoxyribose degradation assay in which hydrogen peroxide is added, more or less create a tissue damage scenario with relatively high ROS concentrations.

ANA and 3-HANA proved as efficient antioxidants in the H_2_O_2_/Fe^III^/ascorbic acid and Fe^III^/ascorbic acid deoxyribose degradation assay variants (Fig. 4a–d). The results of assay variants with or without EDTA addition suggested further that both iron chelation and ROS scavenging may have contributed to the observed effect. No pro-oxidant effects were observed, which can be interpreted that ANA and 3-HANA were not able to substitute the function of ascorbic acid in the Fe^III^ or H_2_O_2_/Fe^III^ assay variants (data not shown). In contrast, Iwahashi et al. observed that 3-HANA increased hydroxyl radical production in reaction mixtures containing iron(III) and hydrogen peroxide or superoxide dismutase; the concentrations of hydroxyl radicals were determined by EPR (electron paramagnetic resonance) [14]. Furthermore, various authors have provided evidence that 3-HANA can reduce iron(III) to iron(II) in phosphate buffer solution of pH 7.4 [14], [15]. These contradictions in the results can be explained by the different experimental arrangements that were used by the various authors. Moreover, the other authors did not explore oxidative degradation of 2-deoxy-d-ribose such as it is the case in this study. In the iron(III) reduction assay, they employed a spectrophotometric determination of iron(II) concentrations by iron(II) chelators, bathophenanthroline disulfonic acid or phenanthroline [14], [15]. It cannot be ruled out that the used iron(II) chelators could have shifted the reaction equilibrium towards iron reduction [25].

**Fig. 5:**
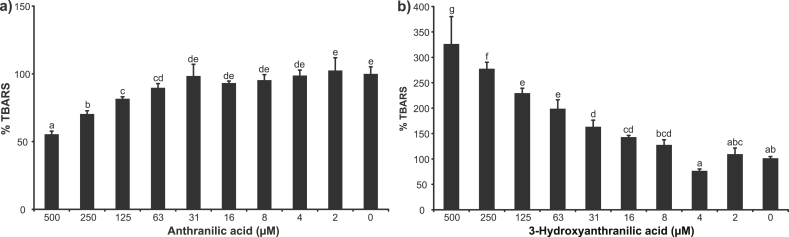
Effects of anthranilic acid and 3-hydroxyanthranilic acid on generation of thiobarbituric acid reactive species in iron(II) autoxidation assay. Error bars indicate the standard deviation of three replicates; letters (a–g) indicate different levels of significance (95% Duncan).

To clarify the contradictions, we performed an iron(II) autoxidation assay [18], [19]. In this assay, ANA and 3-HANA showed different effects (Fig. 5). Whereas ANA inhibited generation of TBARS in a concentration range of 63–500 μM (Fig. 5a), 3-HANA showed apparent pro-oxidant activity between the concentrations of 31 and 500 μM (Fig. 5b). Iron(II) is oxidized by molecular oxygen which is reduced to superoxide anion radical. This radical can spontaneously dismutate or be reduced to hydrogen peroxide. Then, hydrogen peroxide starts the Fenton reaction and hydroxyl radical production. Hydroxyl radicals are detected as TBARS (2-deoxy-d-ribose decomposition products). In this assay, iron does not require to be reduced before participation in the Fenton reaction [19]. The test compound can influence TBARS generation by affecting of iron(II) autoxidation rate and/or by ROS scavenging.

Both mechanisms can contribute to the antioxidant activity of ANA. If iron is a central atom in coordination complexes with ANA, the redox potential of iron is shifted into the anodic direction as shown by differential pulse voltammetry. An anodic shift decreases the rate of iron(II) autoxidation. Furthermore, the 2-amino group can also contribute to the antioxidant effect of ANA by hydroxyl radical scavenging.

The pro-oxidant effect of 3-HANA in the iron(II) autoxidation assay can be explained by semiquinone formation. The hydroxyl group of 3-HANA can be oxidized to a semiquinone by superoxide anion radical, a reaction product of iron(II) autoxidation. Semiquinones and their derivatives are potent reducing agents which can reduce molecular oxygen to superoxide anion radical [26] that can dismutate to hydrogen peroxide. The dismutation can occur either spontaneously or catalyzed by iron–3-HANA complexes [27]. Superoxide anion radical may be reduced also to hydrogen peroxide by a further molecule of 3-HANA. Both processes, dismutation and reduction of superoxide radical, lead to quick formation of hydrogen peroxide which enters in the Fenton reaction generating hydroxyl radicals.

**Fig. 6:**
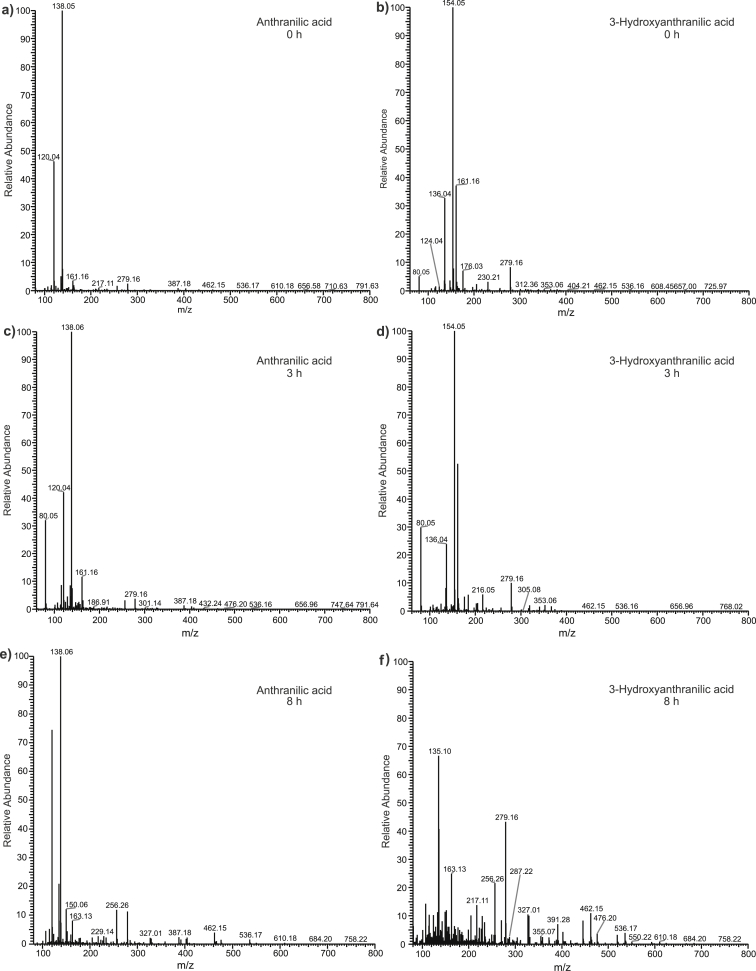
ESI–MS spectra in the positive mode of anthranilic acid (ANA) and 3-hydroxyanthranilic acid (3-HANA) degradation in the presence of iron(II) and hydrogen peroxide.

The nano-ESI–MS analyses of ANA and 3-HANA oxidation stability confirmed higher redox reactivity of the latter compared to the former (Fig. 6). The exploration of oxidation stability was carried out in the Fenton reaction mixture containing hydrogen peroxide and iron(II). The reaction mixtures were analyzed after reaction times of 3 and 8 h by direct infusion. After 3 h, the ions of both tested acids were detectable (Fig. 6 c,d). After 8 h, the 3-HANA ion had vanished from the mass spectrum and numerous new ions with lower and higher masses appeared. By contrast, ANA was still present in the analyzed solution (Fig. 6 e,f). In addition, the mass spectrum of 3-HANA that was recorded after 8 h incubation showed more ions of reaction products than of the ANA ion itself.

Changing ANA and 3-HANA concentrations as well as their actual ratio in tissues can modulate T-cell activity in the immune system. 3-HANA can also affect various inflammatory factors [7], [28]. A decreasing concentration ratio 3-HANA:ANA was detected in brains of patients suffering from some neurological diseases such as stroke, chronic brain injury and Huntington's disease [6], [7]. The altered levels of 3-HANA can lead to modified inflammatory and immune system responses often associated with the above-mentioned diseases. Several hypotheses propose causes for this decreasing ratio, but none of them explains fully all aspects of the phenomenon. One of many possible mechanisms, which could lead to decreasing 3-HANA concentrations, is the reaction of 3-HANA with hydroxyl radical under oxidative stress conditions. 3-HANA is more susceptible to oxidation by ROS than ANA. The production of hydroxyl radicals in brains of patients can be enhanced by accumulation of transition metals, especially iron [8], [29], which initializes the Fenton reaction.

## Conclusions

Both, ANA and 3-HANA, are redox active and can act as antioxidants in certain chemical environments. Furthermore they form coordination complexes with iron. A combination of differential pulse voltammetry, deoxyribose degradation assay, iron(II)-autoxidation assay and direct infusion ESI–MS of coordination complexes and oxidation reaction solutions succeeded in revealing some minor but important differences in the redox chemistry of these two tryptophan metabolites. 3-HANA is much more instable than ANA when attacked by hydroxyl radicals in an oxidative stress scenario. The high redox reactivity of 3-HANA, especially in presence of iron(II), concurs with the observation that patients suffering from various neurological diseases (e.g. stroke, chronic brain injury and Huntington's disease) show decreased levels of this metabolite.
